# Molecular analysis of pediatric brain tumors identifies microRNAs in pilocytic astrocytomas that target the MAPK and NF-κB pathways

**DOI:** 10.1186/s40478-015-0266-3

**Published:** 2015-12-18

**Authors:** Tania A. Jones, Jennie N. Jeyapalan, Tim Forshew, Ruth G. Tatevossian, Andrew R. J. Lawson, Sheena N. Patel, Gabriel T. Doctor, Muhammad A. Mumin, Simon R. Picker, Kim P. Phipps, Antony Michalski, Thomas S. Jacques, Denise Sheer

**Affiliations:** Centre for Genomics and Child Health, Blizard Institute, Barts and The London School of Medicine and Dentistry, Queen Mary University of London, 4 Newark Street, London, E1 2AT UK; Department of Histopathology, Great Ormond Street Hospital for Children NHS Trust, London, WC1N 3JH UK; Developmental Biology and Cancer Programme, UCL-Institute of Child Health, University College London, London, WC1N 1EH UK; Department of Neurosurgery, Great Ormond Street Hospital for Children NHS Trust, London, WC1N 3JH UK; Department of Oncology, Great Ormond Street Hospital for Children NHS Trust, London, WC1N 3JH UK; Present address: Research Department of Pathology, Cancer Institute, University College London, 72 Huntley St, Camden Town, London, WC1E 6DD UK; Present address: Department of Neuropathology, St Jude Children’s Research Hospital, Memphis, TN 38105 USA; Present address: Inivata Limited, Li Ka Shing Centre, Cambridge, CB2 0RE UK

**Keywords:** Grade I astrocytoma, Children, Senescence-associated secretory phenotype (SASP), miR-146a, miR-155, Neuroinflammation, *KIAA1549-BRAF*

## Abstract

**Introduction:**

Pilocytic astrocytomas are slow-growing tumors that usually occur in the cerebellum or in the midline along the hypothalamic/optic pathways. The most common genetic alterations in pilocytic astrocytomas activate the ERK/MAPK signal transduction pathway, which is a major driver of proliferation but is also believed to induce senescence in these tumors. Here, we have conducted a detailed investigation of microRNA and gene expression, together with pathway analysis, to improve our understanding of the regulatory mechanisms in pilocytic astrocytomas.

**Results:**

Pilocytic astrocytomas were found to have distinctive microRNA and gene expression profiles compared to normal brain tissue and a selection of other pediatric brain tumors. Several microRNAs found to be up-regulated in pilocytic astrocytomas are predicted to target the ERK/MAPK and NF-κB signaling pathways as well as genes involved in senescence-associated inflammation and cell cycle control. Furthermore, IGFBP7 and CEBPB, which are transcriptional inducers of the senescence-associated secretory phenotype (SASP), were also up-regulated together with the markers of senescence and inflammation, *CDKN1A (p21), CDKN2A (p16)* and *IL1B.*

**Conclusion:**

These findings provide further evidence of a senescent phenotype in pilocytic astrocytomas. In addition, they suggest that the ERK/MAPK pathway, which is considered the major driver of these tumors, is regulated not only by genetic aberrations but also by microRNAs.

**Electronic supplementary material:**

The online version of this article (doi:10.1186/s40478-015-0266-3) contains supplementary material, which is available to authorized users.

## Introduction

Pilocytic astrocytomas (WHO grade I) are the most common central nervous system tumors in the 5 to 19 year age group. They are cystic, well-circumscribed tumors, which rarely progress and can usually be removed using surgery [9]. This accounts for their having a more favourable prognosis than diffuse and other infiltrative astrocytomas [39]. Molecular studies on pilocytic astrocytomas have identified recurrent *BRAF* gene fusions and other alterations that activate the ERK/MAPK signal transduction pathway [15, 27, 56]. Activated ERK/MAPK signaling is believed to drive cellular proliferation and then to trigger senescence, giving rise to the indolent phenotype of pilocytic astrocytomas [24, 42].

MicroRNAs are short RNA molecules that modulate gene expression post-transcriptionally by repressing translation or degrading the mRNA transcript [26]. They regulate numerous biological processes including cell proliferation and differentiation, and have major roles in embryogenesis, including brain and spinal cord development and neurogenesis [13, 41]. Many microRNAs are involved in the initiation and progression of cancer [3, 37], and recent studies of pediatric brain tumors have identified tumor-specific microRNA signatures [5, 21, 38].

To further our knowledge of the molecular drivers of pilocytic astrocytomas, we conducted a detailed investigation of microRNA and gene expression in these tumors, and characterised the findings by pathway analysis. Our results revealed a distinctive microRNA and gene expression profile in pilocytic astrocytoma compared to other pediatric brain tumors. We found that many predicted targets of up-regulated microRNAs in pilocytic astrocytomas are known regulators of the ERK/MAPK and NF-κB pathways. These findings suggest an important modulatory role for microRNAs in critical pathways involved in pilocytic astrocytomas.

## Materials and methods

### Tumor samples and controls

miRNA and mRNA profiling was performed on a range of fresh frozen brain tumor samples (the *test* cohort) from 57 children, aged 1–16 years, and on 7 normal adult brain controls (4 frontal lobe and 3 cerebellum, Biochain) (Table 1a). A further 13 pediatric brain tumor samples were used as a *validation* cohort (Table 1b). Tumors were classified using criteria defined by the World Health Organization (WHO) [36]. Access to samples and clinical data complied with Local Research Ethics Committee (LREC) regulations: Great Ormond Street Hospital LREC reference number 05/Q0508/153. All the *test* pilocytic astrocytomas were shown previously to contain the *KIAA1549-BRAF* gene fusion [32]. The *BRAF* V600E mutation was also examined in the high-grade astrocytomas in the *test* cohort. *KIAA1549-BRAF* fusion and the *BRAF* V600E mutations were also tested in the *validation* cohort using PCR according to published protocols [32, 45].

**Table 1:** Pediatric tumor cohorts

Tumor	Pathology	Grade	Location	Sex	Age	BRAF status
a.						
PGNT	Papillary glioneuronal	I	Frontal lobe	M	14	nd
PA1	Pilocytic astrocytoma	I	Posterior fossa	F	16	*KIAA1549-BRAF* exon 16-exon 9
PA2	Pilocytic astrocytoma	I	Posterior fossa	M	12	*KIAA1549-BRAF* exon 16-exon 9
PA3	Pilocytic astrocytoma	I	Posterior fossa	M	14	*KIAA1549-BRAF* exon 16-exon 9
PA5	Pilocytic astrocytoma	I	Posterior fossa	F	3	*KIAA1549-BRAF* exon 16-exon 9
PA6	Pilocytic astrocytoma	I	Cerebellum	F	8	*KIAA1549-BRAF* exon 15-exon 9
PA7	Pilocytic astrocytoma	I	Cerebellum	M	1	*KIAA1549-BRAF* exon 16-exon 11
PA8	Pilocytic astrocytoma	I	Cerebellum	M	1	*KIAA1549-BRAF* exon 16-exon 9
PA9	Pilocytic astrocytoma	I	Cerebellum	F	2	*KIAA1549-BRAF* exon 16-exon 11
PA10	Pilocytic astrocytoma	I	Cerebellum	M	2	*KIAA1549-BRAF* exon 16-exon 9
PA11	Pilocytic astrocytoma	I	Cerebellum	M	6	*KIAA1549-BRAF* exon 16-exon 9
PA12	Pilocytic astrocytoma	I	Cerebellum	F	11	*KIAA1549-BRAF* exon 15-exon 9
PA13	Pilocytic astrocytoma	I	Posterior fossa	M	4	*KIAA1549-BRAF* exon 16-exon 9
PA14	Pilocytic astrocytoma	I	Posterior fossa	F	6	*KIAA1549-BRAF* exon 16-exon 9
PA15	Pilocytic astrocytoma	I	Posterior fossa	M	9	*KIAA1549-BRAF* exon 16-exon 9
DA1	Diffuse astrocytoma	II	Temporal lobe	F	12	No *BRAFV600E*
DA2	Diffuse astrocytoma	II	Unknown	F	14	No *BRAFV600E*
DA3	Diffuse astrocytoma	II	Occipital lobe	M	4	nd
DA4 *	Diffuse astrocytoma	II	Unknown	M	17	*BRAFV600E* present
AA1	Anaplastic astrocytoma	III	Conus medullaris	M	15	nd
AA2	Anaplastic astrocytoma	III	Temporal lobe	F	3	No *BRAFV600E*
AA3 *	Anaplastic astrocytoma	III	Unknown	F	11	No *BRAFV600E*
GBM1	Glioblastoma	IV	Frontal lobe	F	15	No *BRAFV600E*
GBM2	Glioblastoma	IV	Temporoparietal	M	10	No *BRAFV600E*
GBM3	Glioblastoma	IV	Brainstem	F	4	nd
GBM4	Glioblastoma	IV	Left frontal lobe	F	10	No *BRAFV600E*
GBM5	Glioblastoma	IV	Spinal cord	F	7	nd
E1	Ependymoma	III	Posterior fossa	M	4	
E2	Ependymoma	III	Parietal lobe	F	6	
E3	Ependymoma	II	Posterior fossa	F	2	
E4	Ependymoma	III	Posterior fossa	F	2	
E5	Ependymoma	II	Temporal lobe	M	12	
E6	Ependymoma	III	Posterior fossa	M	6	
E7	Ependymoma	III	Posterior fossa	M	2	
E8	Ependymoma	II	Supratentorial (frontal)	M	14	
E9	Ependymoma	II	Posterior fossa	M	3	
E10	Ependymoma	II	Posterior fossa	M	4	
E11	Ependymoma	III	Posterior fossa	F	nd	
E12	Ependymoma	III	Fourth ventricle	M	1	
E13	Ependymoma	II	Spinal seed	F	6	
E14	Ependymoma	III	Posterior fossa	F	6	
M1	Medulloblastoma	IV	Posterior fossa	M	4	
M2	Medulloblastoma	IV	Posterior fossa	M	1	
M3	Medulloblastoma	IV	Posterior fossa	F	6 months	
M4	Medulloblastoma	IV	Posterior fossa	M	7	
M5	Medulloblastoma	IV	Posterior fossa	M	3	
M6	Medulloblastoma	IV	Posterior fossa	M	9	
M7	Medulloblastoma	IV	Posterior fossa	M	7	
M8	Medulloblastoma	IV	Posterior fossa	M	9	
M9	Medulloblastoma	IV	Posterior fossa	M	4	
M10 *	Medulloblastoma	IV	Posterior fossa	F	5	
R1	Atypical Rhabdoid	IV	Brainstem	M	1	
R2	Atypical Rhabdoid	IV	Posterior fossa	F	7 months	
R3	Atypical Rhabdoid	IV	Supratentorial	F	2	
R4	Atypical Rhabdoid	IV	Posterior fossa	M	9 months	
R5	Atypical Rhabdoid	IV	Posterior fossa	F	2 months	
P1	Choroid Plexus Papilloma	I	Intraventricular (frontal lateral ventricle)	M	10 months	
P2	Choroid Plexus Papilloma	I	Posterior fossa	M	1	
P3	Choroid Plexus Papilloma	I	Left lateral ventricle	M	3	
P4	Choroid Plexus Papilloma	I	Unknown	F	nd	
AC1	Adult control cerebellum		Cerebellum	M	22	
AC2	Adult control frontal lobe		Frontal lobe	M	41	
AC3	Adult control cerebellum		Cerebellum	M	21	
AC4	Adult control cerebellum		Cerebellum	M	26	
AC5	Adult control frontal lobe		Frontal lobe	M	82	
AC6	Adult control frontal lobe		Frontal lobe	M	25	
AC7	Adult control frontal lobe		Frontal lobe	M	27	
b.						
PA16	Pilocytic astrocytoma	I	Posterior fossa	M	9	nd
PA17	Pilocytic astrocytoma	I	Posterior fossa	M	12	nd
PA18	Pilocytic astrocytoma	I	Suprasellar	M	3	*KIAA1549-BRAF* exon 15-exon 9
PA19	Pilocytic astrocytoma	I	3^rd^ ventricle	M	16	nd
PA20	Pilocytic astrocytoma	I	Brain stem	F	3	*KIAA1549-BRAF* exon 15-exon 9
PA21	Pilocytic astrocytoma	I	Posterior fossa	F	4	*KIAA1549-BRAF* exon 16-exon 9
PA22	Pilocytic astrocytoma	I	Posterior fossa	M	16	*KIAA1549-BRAF* exon 16-exon 9
PA23	Pilocytic astrocytoma	I	Posterior fossa	F	2	*KIAA1549-BRAF* exon 16-exon 11
PA24	Pilocytic astrocytoma	I	Posterior fossa	F	12	*KIAA1549-BRAF* exon 16-exon 9
GBM6	Glioblastoma	IV	Midbrain/Thalamus	F	14	-
GBM7	Glioblastoma	IV	Occipital lobe	M	4 months	-
GBM8	Glioblastoma	IV	Thalamic/intraventriclar	F	10	-
GBM9	Glioblastoma	II	Thalamic	M	15	-

(a) Test cohort analysed using Illumina MicroRNA Expression Arrays (MI-v2) and Illumina HumanHT-12_v3 Beadchip system, with the exception of samples marked with an asterisk (*), that were used for validation only

(b) Validation cohort used for confirmation of differentially expressed microRNAs and genes. Annotation: − negative, *nd* not determined

### MicroRNA and gene expression arrays

MicroRNA expression was profiled in the *test* tumor cohort and the normal brain controls using Illumina MicroRNA Expression Arrays (MI-v2). This system screens 1,146 known and putative microRNAs including 97 % of microRNAs in miRBase (Release 12.0). Gene expression profiles were obtained for the same tumors and controls using the Illumina HumanHT-12_v3 Beadchip system. RNA was extracted from the samples using TRIzol (Invitrogen) and eluted into RNAse-free water using the RNeasy mini kit (Qiagen). cDNA was synthesized using random hexamers and the SuperScript First-Strand cDNA synthesis system (Invitrogen). Hybridization and scanning were performed in-house at the Barts and The London Genome Centre.

### Statistical analysis

The microRNA and gene expression-averaged tumor profiles were compared to normal adult brain controls using GenomeStudio and GeneSpring Multi-Omic Analysis v12.1 software. Data were subjected to thresholding, log transformation (log2), normalization (quantile) and baseline transformation (median to all samples). Normalized data of microRNA and gene expression are shown in Additional file 1: Table S1 and Additional file 2: Table S2. All data were deposited in NCBI’s Gene Expression Omnibus [12] (http://www.ncbi.nlm.nih.gov/geo) and are accessible through GEO accession number GSE42658. Differential expression was defined as fold change (FC) > 2 with FDR corrected (Benjamini Hochberg) *p-*values < 0.05. Unsupervised hierarchical clustering (Euclidean method), was performed on both the microRNA and mRNA expression data. The differentially expressed microRNAs and genes are listed in Additional file 3: Table S3 and Additional file 4: Table S4 for tumor groups with more than three samples.

### Pathway analysis

Ingenuity Pathway Analysis (Ingenuity Systems Inc., Redwood City, USA) was used to identify over-represented pathways for significantly differentially expressed genes in pilocytic astrocytomas. The significance value associated with the functional analysis is expressed as a *p-*value calculated by comparing the number of differentially expressed genes that participate in a given function or pathway, relative to the total number of occurrences of these proteins in all functional/pathway annotations stored in Ingenuity Pathway Knowledge Base. Multiple-testing corrected *p-*values were calculated using the Benjamini-Hochberg method. For each functional annotation, a statistical quantity is calculated called the regulation z-score. The purpose of the z-score is to identify increased or decreased biological functions that are likely implicated by the observed gene expression changes. Hence, the *p-*values measure the observed and predicted regulated gene sets, and the z-score assesses the match of the observed and predicted up/down regulation patterns. For this study, we only considered a predicted activation/inhibition status as significant if the *p-*value was < +/−0.05 and the z-score > 2.0 or < 2.0 respectively.

### Taqman microRNA assays

TaqMan miRNA assays were used to validate differentially expressed microRNAs of interest. Control microRNAs RNU48 and miR-423-3p were tested for stability over all tumors and miR-423-3p was selected as the most stable control. Hence all samples were normalized to miR-423-3p and fold change calculated relative to the average expression in adult cerebellum and frontal lobe (BioChain, A508112 and B210079 respectively). Differential expression was validated for miR-542-3p, miR-503, miR-146a, miR-34a, miR-155, miR-124*, miR-129 and miR-129*. TargetScan Release 6.2 (http://www.targetscan.org) was used to search for predicted targets of microRNAs of interest. MicroRNAs are named using the “miR’ prefix and a unique identifying number which is assigned sequentially [2]. Identical or very similar miRNA sequences within a species can be given the same number, with their genes distinguished by a letter and/or numerical suffixes (e.g. miR-450a and miR-450b are slightly different in sequence, whereas those of miR-450a-1 and miR-450a-2 are identical). Some miRNA hairpin precursors give rise to two excised miRNAs, one from each arm. Previous annotations used the nomenclature miR-124 and miR-124* for the guide and passenger strand respectively, and some of these names were included in our Illumina MicroRNA Expression Arrays (MI-v2). However, the mature sequences derived from both arms of the hairpin may be biologically functional, so current nomenclature uses miR-542-5p and miR-542-3p to designate miRNAs from the ‘5’ and ‘3’ arms respectively, [18, 29].

### RT-qPCR

PCR primers were designed using Primer 3 software (http://frodo.wi.mit.edu/primer3/) (Additional file 5: Table S5). RT-qPCR was used to quantify the levels of mRNA expression for selected genes, using SYBR Green JumpStart Taq ReadyMix kit (Sigma-Aldrich), 50 ng cDNA, and 0.1 μM primers in a reaction volume of 20 μl. Assays were run on an ABI 7500 Real Time PCR System (ABI). PCR analyses were conducted in triplicate for each sample and melting curves analyzed for correct product size. Control genes *TBP* and *CREB1* were tested for stability over all tumors, and *TBP* selected as the most stable endogenous control. Therefore, all samples were normalized to *TBP* and fold change calculated relative to the average expression in adult cerebellum (BioChain, A508112), and frontal lobe (BioChain B210079). Relative quantification was calculated using the 2-ddC_t_ method [35].

## Results

### Characterization of the pediatric brain tumor cohort

**Fig. 1:**
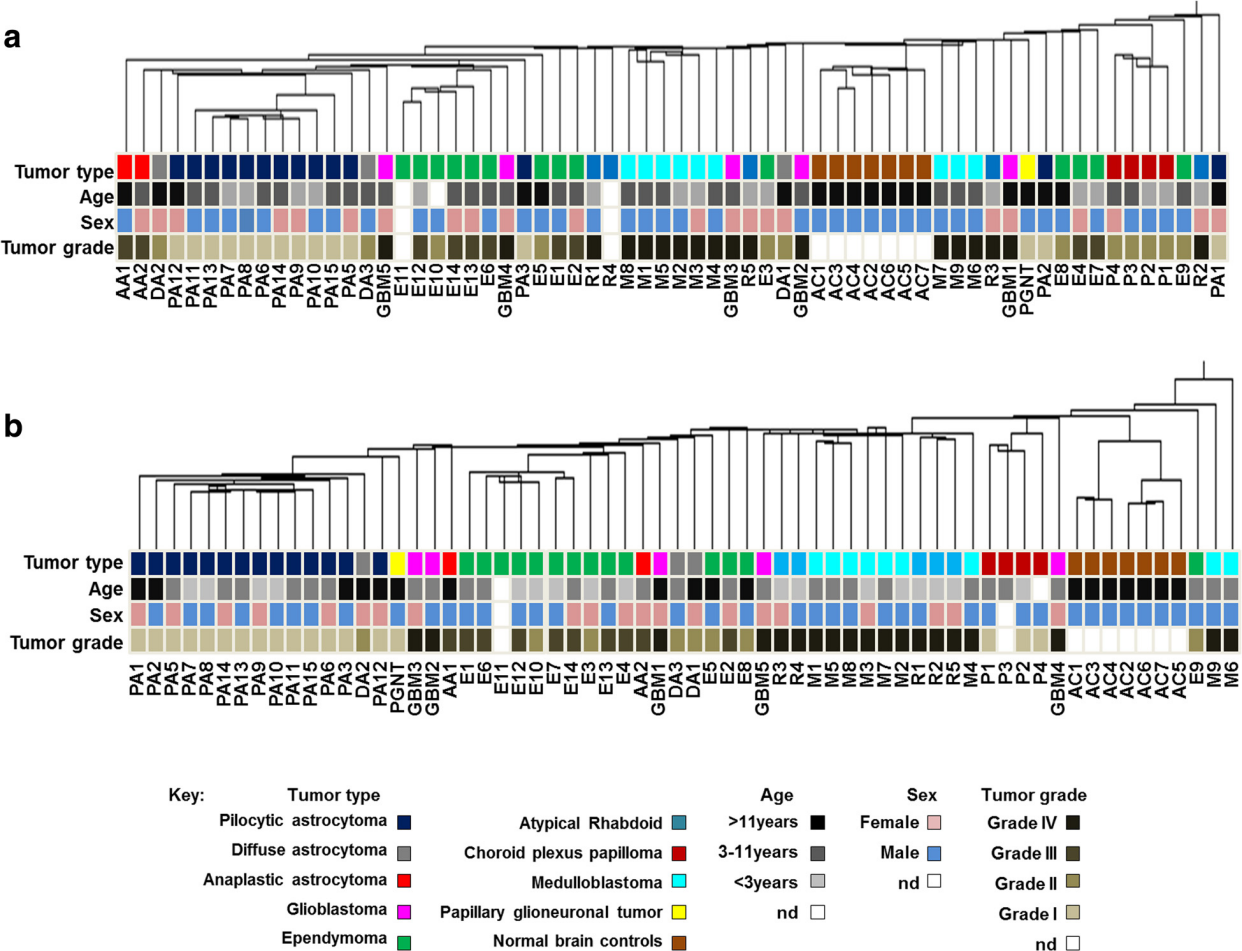
Hierarchical clustering of microRNA and mRNA expression stratifies tumor subgroups. Unsupervised hierarchal clustering (Euclidean method) was performed on (**a**) microRNA and (**b**) mRNA expression data. Tumors represented as: PA-pilocytic astrocytoma, PGNT-papillary glioneuronal tumor, DA-diffuse astrocytoma, AA-anaplastic astrocytoma, GBM-glioblastoma, E-ependymoma, M-medulloblastoma, R-atypical rhabdoid tumor, P-choroid plexus papilloma and AC1, AC3, AC4-adult control cerebellum and AC2, AC5, AC6, AC7-adult control frontal lobe. nd – not determined

We first examined microRNA and gene expression in the *test* tumor cohort that consisted of a papillary glioneuronal tumor (*n* = 1), pilocytic astrocytomas (*n* = 14), diffuse astrocytomas (*n* = 3), anaplastic astrocytomas (*n* = 2), glioblastomas (*n* = 5), atypical teratoid rhabdoid tumors (AT/RT) (*n* = 5), medulloblastomas (*n* = 9), ependymomas (*n* = 14) and choroid plexus papillomas (*n* = 4), total = 57 samples (Table 1a). Differential microRNA expression across the averaged tumor groups was calculated to obtain fold changes compared to normal adult brain controls (FDR < 0.05, Additional file 3: Table S3). The number of up- and down-regulated microRNAs for each tumor type is shown in Table 2. Unsupervised hierarchical clustering (Euclidean method) of microRNAs clustered the tumors by pathology in the low-grade tumors, and showed higher-grade tumors to be more widely distributed (Fig. 1a). A similar pattern was also found using gene expression data (Additional file 2: Table S2), with higher-grade tumors showing greater heterogeneity than low-grade tumors (Fig. 1b). Following initial comparison with other tumor types, microRNA profiles were then investigated in detail in pilocytic astrocytomas, all of which contained the *KIAA1549:BRAF* gene fusion.

**Table 2:** Number of differentially expressed microRNAs in pediatric brain tumors compared to normal adult brain

Pathology	*n*	Regulation		Total
		Up	Down	
Pilocytic astrocytoma	14	42	49	91
Pilocytic astrocytoma^a^	14	27	33	60
Diffuse astrocytoma	3	1	2	3
Anaplastic astrocytoma	2	6	8	14
Glioblastoma	5	0	5	5
Ependymoma	14	60	75	135
Medulloblastoma	9	25	35	60
Medulloblastoma^a^	9	10	29	39
Atypical Teratoid/Rhabdoid tumor	5	15	17	32
Choroid plexus papilloma	4	47	63	110

^a^Pilocytic astrocytomas and medullobastomas were also compared to normal adult cerebellum. Fold change > 2 with a FDR corrected *p*-value < 0.05

### MicroRNA profile and the predicted targeted pathways in Pilocytic Astrocytomas

**Fig. 2:**
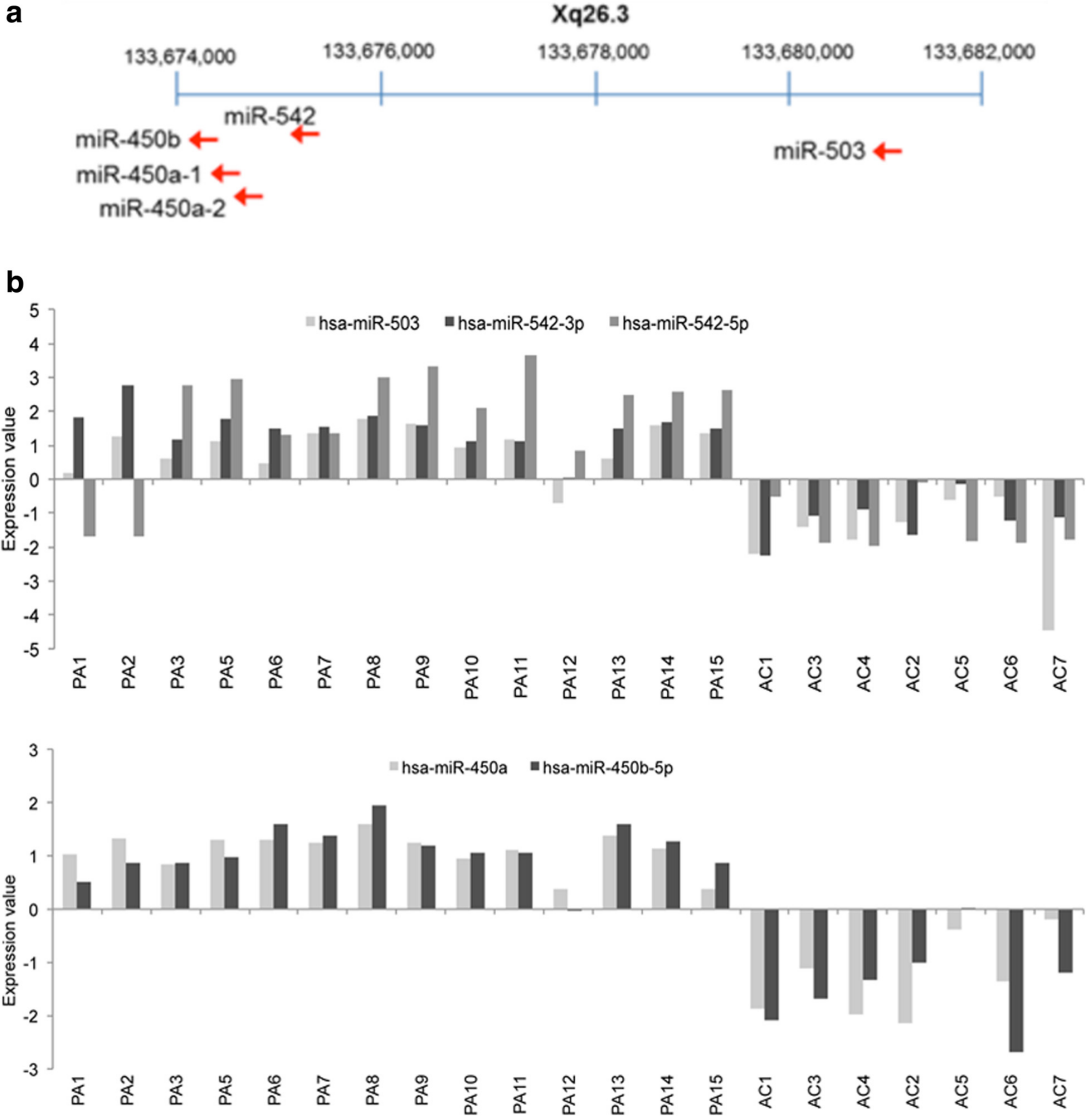
Expression of a cluster of microRNAs on Xq26.3 in pilocytic astrocytomas. (**a**) Map of microRNA genes showing up-regulated microRNAs transcribed in the same direction. (**b**) Illumina MicroRNA Expression array (MI-v2) data showing up-regulation of miR-503, miR-542-3p, miR-542-5p, miR-450a and miR-450b-5p in pilocytic astrocytoma compared to normal brain controls. The Illumina probe miR-450a contains the seed sequence present in both miR-450a-1 and miR-450a-2

Profiles from pilocytic astrocytomas were compared with combined adult brain controls, and also with normal adult cerebellum, since this subset of tumors were all located in the cerebellum (Additional file 3: Table S3). Up- and down-regulated microRNAs compared to normal adult cerebellum are shown in Table 3. Amongst the top up-regulated microRNAs was a cluster located on Xq26.3, consisting of miR-542-5p, miR-542-3p, miR-503, mir-450a and miR-450b-5p (Fig. 2). The results were not gender-biased. Other top up-regulated microRNAs included miR-224, miR-146a, miR-34a and the miR-106a ~ miR-363 cluster (Table 3).

**Table 3:** The top deregulated microRNAs in pilocytic astrocytoma compared to normal adult cerebellum

TargetID	p (Corr)	FC	Log FC
a.			
hsa-miR-224	0.00632	10.09	3.33
hsa-miR-542-5p	0.00558	9.69	3.28
hsa-miR-542-3p	0.02366	7.51	2.91
hsa-miR-450b-5p	0.00285	6.89	2.78
hsa-miR-503	0.00120	6.80	2.76
hsa-miR-450a	0.01211	6.67	2.74
hsa-miR-886-5p	0.00551	6.17	2.62
hsa-miR-767-5p	0.00002	4.94	2.31
HS_29	0.01811	4.41	2.14
hsa-miR-708	0.02645	3.92	1.97
hsa-miR-146a	0.01076	3.84	1.94
hsa-miR-34a	0.00268	3.13	1.65
solexa-539-2056	0.04379	3.03	1.60
hsa-miR-335*	0.00486	2.81	1.49
hsa-miR-18b	0.04529	2.79	1.48
hsa-miR-452*	0.00000	2.58	1.37
hsa-miR-18a	0.00441	2.56	1.36
hsa-miR-199a*:9.1	0.03622	2.56	1.36
hsa-miR-574-5p	0.00000	2.39	1.26
hsa-miR-296-3p	0.02907	2.36	1.24
hsa-miR-155	0.01372	2.33	1.22
hsa-miR-24-2*	0.00000	2.33	1.22
hsa-miR-106a	0.00149	2.32	1.21
hsa-miR-21*	0.00073	2.22	1.15
HS_262.1	0.01814	2.16	1.11
hsa-miR-363	0.01021	2.07	1.05
hsa-miR-371-3p	0.00002	2.03	1.02
b.			
hsa-miR-129-3p	0.00062	−44.25	−5.47
hsa-miR-129*	0.00000	−27.08	−4.76
hsa-miR-1224-5p	0.00515	−16.54	−4.05
hsa-miR-124a:9.1	0.00357	−15.84	−3.99
hsa-miR-326	0.01508	−12.16	−3.60
hsa-miR-124	0.00073	−9.66	−3.27
hsa-miR-204	0.00140	−9.65	−3.27
hsa-miR-1296	0.00000	−6.94	−2.80
hsa-miR-885-5p	0.00006	−6.76	−2.76
hsa-miR-128a:9.1	0.00149	−5.78	−2.53
hsa-miR-218	0.00504	−5.72	−2.52
hsa-miR-133b	0.00000	−5.57	−2.48
hsa-miR-874	0.00013	−5.52	−2.46
hsa-miR-769-3p	0.02429	−4.96	−2.31
hsa-miR-485-3p	0.01589	−4.70	−2.23
hsa-miR-128b:9.1	0.00049	−4.68	−2.23
HS_182.1	0.00140	−4.45	−2.15
hsa-miR-107	0.00002	−4.38	−2.13
hsa-miR-383	0.00175	−4.36	−2.12
hsa-miR-656	0.00825	−4.35	−2.12
hsa-miR-124*	0.03622	−4.14	−2.05
hsa-miR-584	0.00130	−3.61	−1.85
hsa-miR-7	0.00108	−3.40	−1.77
hsa-miR-548k	0.01014	−3.39	−1.76
hsa-miR-1237	0.00149	−3.21	−1.68
hsa-miR-299-5p	0.01076	−3.20	−1.68
hsa-miR-1179	0.01508	−2.92	−1.55
hsa-miR-487b	0.03622	−2.75	−1.46
HS_231	0.00367	−2.73	−1.45
hsa-miR-329	0.00101	−2.47	−1.30
hsa-miR-154*	0.00566	−2.47	−1.30
hsa-miR-889	0.01811	−2.15	−1.11
hsa-miR-135a	0.02645	−2.06	−1.04

(a) Up-regulated microRNAs and (b) down-regulated microRNAs. Differential expression was defined as fold change (FC) > 2 with FDR corrected (Benjamini Hochberg) *p-*values <0.05

**Fig. 3:**
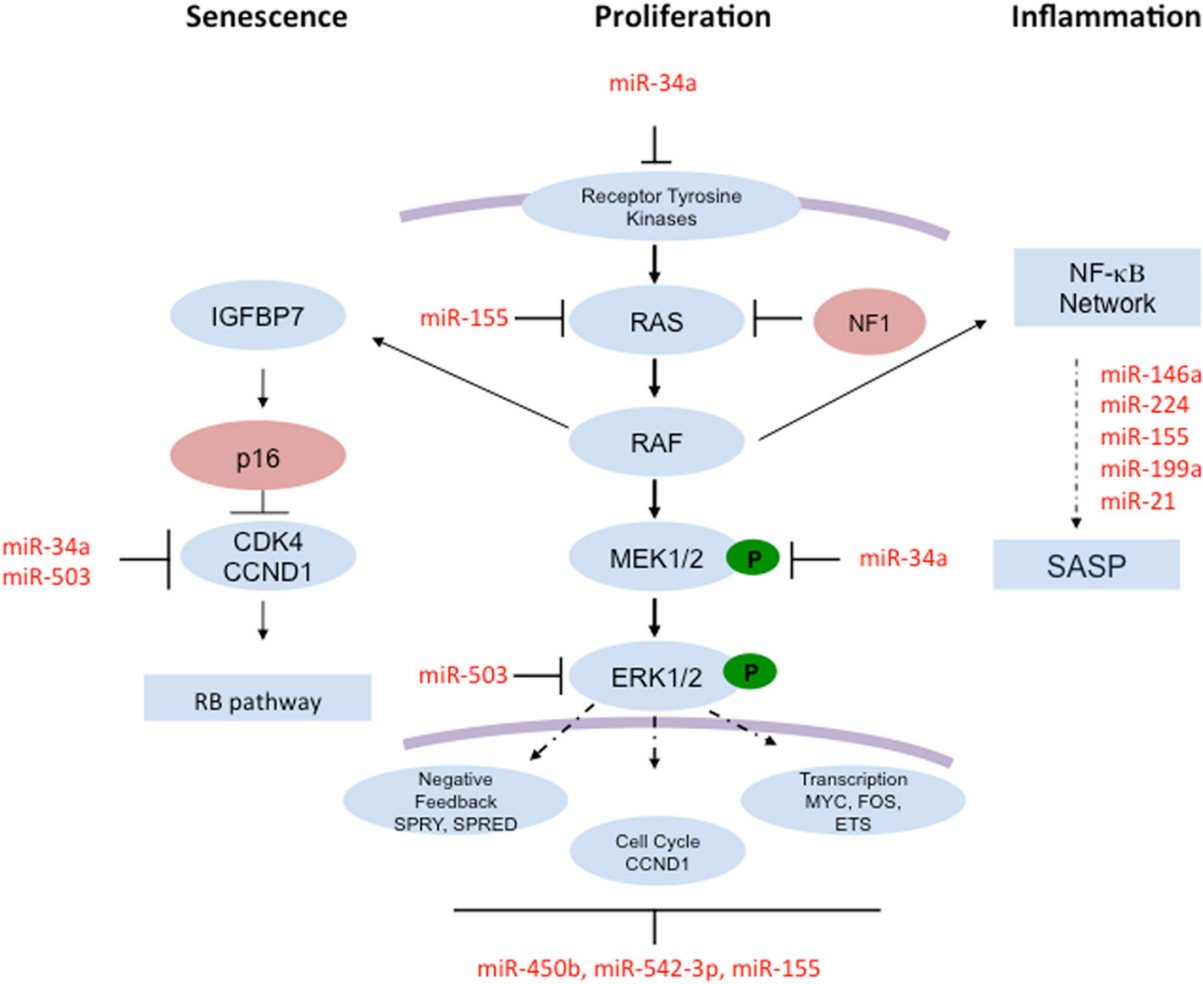
Model showing the interplay of microRNAs in senescence, proliferation and inflammation. MicroRNAs provide negative feedback of the RB pathway, the MAPK pathway and the NF-κΒ network. MiR-34a and miR-503 are predicted to target *CCND1* and *CDK4* to limit cell proliferation via the RB pathway. MiR-155 is predicted to target *KRAS*, miR-34a is predicted to target *MEK1*, whilst down stream targets of the MAPK pathway include *SPRED1* (a predicted target of miR-503), *SPRY1, SPRY2* and *SPRY4* (predicted targets of miR-450b-5p) and *FOS* and *ETS1* (predicted targets of miR-155). MicroRNAs that act as effectors and regulators of NF-κB include miR-146a, miR-155, miR-199a and miR-21. MiR-146a, miR-224 and miR-155 are predicted to target senescenceassociated genes and thus to regulate levels of inflammation

**Fig. 4:**
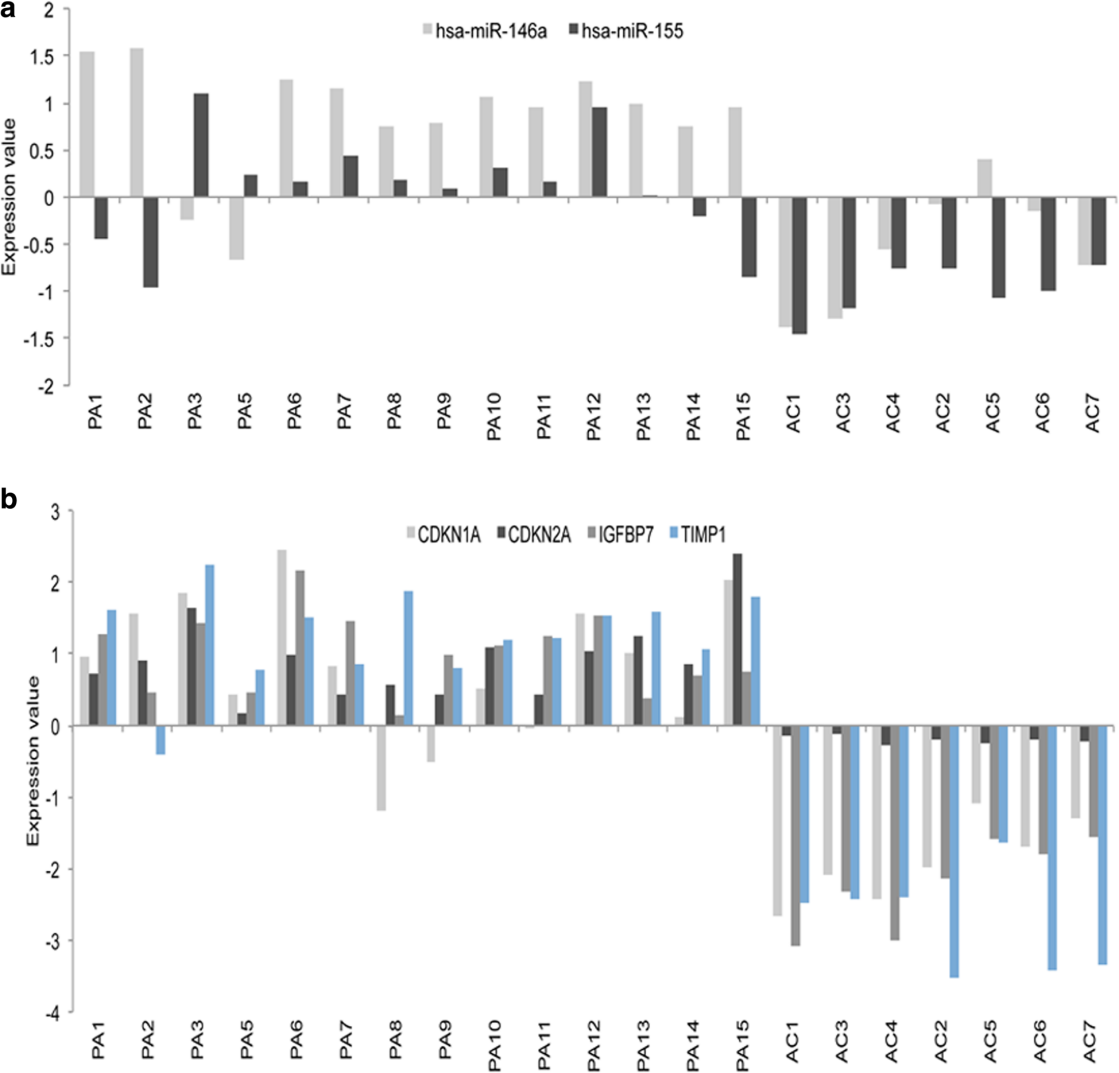
Expression of microRNAs and genes involved in senescence in pilocytic astrocytomas. (**a**) Illumina MicroRNA Expression array (MI-v2) data showing miR-146a and miR-155 are up-regulated in the majority of pilocytic astrocytomas and (**b**) Illumina HumanHT-12_v3 expression array data showing a higher level of expression for *CDKN2A, IGFBP7* and *TIMP1* in pilocytic astrocytomas compared to normal brain controls

All the pilocytic astrocytomas used in the test cohort contain the *KIAA1549-BRAF* fusion that activates the ERK/MAPK pathway. Analysis of up-regulated microRNAs using TargetScan revealed several predicted targets to be regulators of the MAPK pathway. These included miR-155, which targets *KRAS,* miR-34a, which targets *MAP2K1* (*MEK1*), and miR-503, which targets *MAPK1* (*ERK1*) (Fig. 3). Other up-regulated microRNAs, including MiR-450b, miR-542-3p and miR-155, are all predicted to target *ETS1, MYC* and *FOS*, which are regulated by MAPK pathway components. In addition, the up-regulated microRNAs, miR-155, miR-199a, miR-21 and miR-146a (Fig. 4a), target genes within the NF-κB network (Figs. 3). Analysis of brain-specific microRNAs [49] showed no significant differences in expression in pilocytic astrocytomas, apart from down-regulation of miR-218. However, the majority of pilocytic astrocytomas showed low expression of neuronal-specific microRNAs, miR-129, miR-124 and miR-128 [50, 51] (Additional file 3: Table S3).

Oncogene-induced senescence is associated with activation of an inflammatory transcriptome [31], and since senescence markers have been identified in pilocytic astrocytomas, we examined expression levels of microRNAs and genes known to be associated with senescence. MiR-146a was amongst the top up-regulated microRNAs in pilocytic astrocytomas, and is known to modulate senescence-associated inflammatory mediators (Fig. 4a) [4]. Furthermore, *CDKN1A (p21)* and *CDKN2A (p16)* were found to be up-regulated, together with the major transcriptional inducers of senescence-associated secretory phenotype (SASP), *IGFBP7* and *TIMP1* (Fig. 4b)*,* as well as *CEBPB, TIMP2, TIMP3, AXL* and other insulin-like growth factor (IGF)-binding proteins that stimulate inflammation, including *IGFBP2, IGFBP3, IGFBP4* and *IGFBP5* (Additional file 4: Table S4).

Ingenuity Pathway Analysis was used to compare gene expression data between pilocytic astrocytomas and normal adult cerebellum. The top canonical pathways showing significant up-regulation included *Antigen Presentation Pathway, Graft-*versus*-Host Disease Signaling, Type I Diabetes Mellitus Signaling, Hepatic Fibrosis/Hepatic Stellate Cell Activation* and *Dendritic Cell Maturation.* Analysis of the predicted activation state of up-stream transcription factors showed 70 factors predicted as ‘activated’ and 28 predicted as ‘inhibited’ (*p < 0.05,* activation z-score > 2.0) (Additional file 6: Table S6a, b). Predicted ‘activated’ transcription factors included *NF-kB* complex, *TP53*, *IRF7, CEBPB, JUN, RELA* and *CEBPA*, whilst predicted ‘inhibited’ transcription factors included *TRIM24, MYC, MYCN,* estrogen receptor and *RB*.

Using TaqMan assays and Real Time qRT-PCR, we confirmed the differential expression of a set of microRNAs and genes in a selection of the *test* tumor cohort, as well as 13 tumors from the *validation* tumor cohort (Additional file 7: Figure S1 and Additional file 8: Figure S2). We confirmed up-regulation of miR-34a, miR-146a, miR-542-3p, miR-503 and miR-155 and low expression of neuronal microRNAs miR-124*, miR-129 and miR-129* (Additional file 7: Figure S1). Up-regulation of the senescence factors *IGFBP7, CDKN2A (p16), IL6, IL8, CDKN1A (p21)* and down-regulation of *CTCF* was also confirmed (Additional file 8: Figure S2).

## Discussion

To gain a comprehensive understanding of tumorigenesis, it is critical to integrate findings on factors that regulate the transcriptome, such as microRNAs, with genomic analysis. This is especially pertinent in the case of pediatric CNS tumors, which have fewer mutational events than their adult counterparts [48, 54]. MicroRNAs are highly regulated and have essential functions in brain development [23, 30], and certain microRNAs are enriched or specifically expressed in the adult brain [49]. Here, we profiled microRNA and gene expression in a cohort of pediatric brain tumors, and then focused on the potential role of microRNAs in pilocytic astrocytomas.

The mitogen-activated protein kinase (MAPK) signaling cascade is the main molecular pathway that is deregulated in pilocytic astrocytomas [56]. *BRAF*-gene fusions and, in rare cases, *BRAF* V600E mutations give rise to constitutively active *BRAF*, which in turn activates the MAPK pathway. Furthermore, *BRAF* activation has been shown to cause oncogene-induced senescence in vitro [24, 42]. Hence, we obtained gene expression profiles for pilocytic astrocytomas in order to identify any senescent or inflammatory markers that may explain the less aggressive nature of these tumors. Senescence is accompanied by increased secretion of factors involved in senescent signaling. In pilocytic astrocytomas, we observed up-regulation of *IGFBP7* and *CEBPB*, both of which are major transcriptional inducers of SASP-related genes [16]. In addition, we observed up-regulation of *IL1B,* which has been shown to be a SASP-associated protein [10]. Our gene expression profiling of pilocytic astrocytomas also revealed up-regulation of many genes involved in immune function. This was supported by pathway analysis, which showed that pilocytic astrocytomas exhibit a strong immune and inflammatory response.

There is accumulating evidence for a role for certain microRNAs in senescence [1, 17, 19]. The miR-424-miR-503 polycistron, as well as miR-450, miR-542-3p and miR-450b-5p are suggested to play a role in replicative senescence and cellular aging in fibroblasts [6]. MiR-424 and miR-503 can induce G1 arrest in THP-1 cells [14] and may limit proliferation in pilocytic astrocytomas. Recently, expression of miR-542-5p, which is repressed in *MYC*-amplified neuroblastomas was shown to be positively correlated with survival [46]. Interestingly, *CTCF*, a predicted target of miR-542-3p, is down-regulated in pilocytic astrocytoma, and a recent investigation into the higher-order chromatin structure of the *INK4/ARF* locus in senescent cells, has revealed down-regulation of *CTCF* in oncogene-induced senescent cells [20].

Predicted targets of differentially regulated microRNAs frequently included components of the ERK/MAPK and NF-κB signaling pathways (Fig. 3). MiR-503 and miR155 are predicted to target components of the ERK/MAPK pathway, as well as miR-34a which can induce cell cycle arrest by targeting Notch ligand delta-like 1, a component of *NOTCH* signaling [11], and cell cycle regulators *CCND1* and *CDK6* [52]. The same microRNAs are also predicted to target components of NF-κB signaling. MiR-155 has many target genes including *IKKε,* suggesting a role in the immune response [53]. In ovarian tumors, miR-199a negatively regulates *IKKβ* expression to reduce *NF-κB* activity [8]. *NF-κB* is reported to be a direct transcriptional regulator of miR-224 [47] and we observe up-regulation of the miR-224 in our pilocytic astrocytomas. In addition, miR-224 has been shown to target the tumor suppressor *RKIP* in human breast cancer [22]. MiR-146a, which is induced by inflammation in the presence of *NF-κB* and *IL1* receptor signaling to modulate senescent mediators, is up-regulated specifically in pilocytic astrocytomas [4]. In fibroblasts, miR-146a/b down-regulates *IRAK1* to reduce *IL6* and *IL8* secretion in an elegant feedback loop [4]. The expression of miR-146a is a late manifestation of senescence [43] and it is possible that the few pilocytic astrocytomas that do not show elevated expression of miR-146a have not reached a “mature” level of the senescent phenotype.

Brain tissues are composed of multiple neuronal, astroglial, microglial and other cell types, so the relative contributions of each brain cell type to the overall microRNA and gene expression profiles remain to be elucidated. MiR-146a and miR-155 are expressed by activated microglia and astroglial cells in culture [7, 34, 44]. Therefore, it is possible that some of our up-regulated microRNAs could also potentially represent the proliferating microglia that are reported to be present in pilocytic astrocytomas [28]. A recent study has demonstrated that increased activation of RAS/ERK signaling in neocortical progenitors produced a *NEUROG2-ASCL1* switch, which promotes glial fate [55]. This developmental neurogenic to gliogenic switch in transcription factor expression is supported in studies of RAS-driven glioma formation from neural stem cells [25, 40]. In a recently developed mouse model of gliomagenesis, high RAS/ERK levels were found to modify *Ascl1* activity giving rise to glial tumors [33], and we observe clear up-regulation of *ASCL1* in the pilocytic astrocytomas (Additional file 4: Table S4). These authors demonstrated that whilst RAS/ERK signaling is critical for neurodevelopment, *ERK* is the key effector as it phosphorylates the ASCL1 protein resulting in its gliogenic effect. Hence, enhanced RAS/ERK signaling in the pilocytic astrocytomas by activated BRAF may have triggered a gliogenic switch, and this is reflected in the expression of microRNAs that are usually observed in astroglial cells.

## Conclusions

In summary, we show that pilocytic astrocytomas differentially express microRNAs that target genes that encode regulators of the MAPK and NF-κB pathways, as well as genes that are markers of inflammation. These microRNAs and the post-translational regulation of their gene targets may therefore play a role in the unique phenotype of pilocytic astrocytomas.

## Additional files

Additional file 1: Table S1. Normalized microRNA expression data. (XLSX 841 kb) Additional file 2: Table S2. Normalized gene expression data. (XLSX 37339 kb) Additional file 3: Table S3. Differentially expressed microRNAs in pediatric brain tumors (tumor groups with > 3 samples). (XLSX 75 kb) Additional file 4: Table S4. Differentially expressed genes in pediatric brain tumors (tumor groups with > 3 samples). (XLSX 852 kb) Additional file 5: Table S5. (a) List of primer pairs selected for Illumina microRNA expression array (MI-v2) validation. (b) List of primer pairs used to identify *KIAA1549-BRAF* fusion and the *BRAF V600E* mutation. (PDF 58 kb) Additional file 6: Table S6. Ingenuity Pathway Analysis of differentially expressed genes in pilocytic astrocytomas compared to normal adult cerebellum, showing transcription factors with a predicted activation state as (a) ‘activated’ and (b) ‘inhibited’. (PDF 68 kb) Additional file 7: Figure S1. RT-qPCR confirms (a) up-regulation of miR-34a, miR-146a, miR-542-3p and miR-503 in pilocytic astrocytomas. (b) low expression of miR-124*, miR-129 and miR-129* in pilocytic astrocytomas. Relative expression shown as Log_2_ fold change compared to normal adult cerebellum and frontal lobe (normalized to miR-423-3p). Data represent two technical replicates ± SD. (ZIP 516 kb) Additional file 8: Figure S2. RT-qPCR confirms (a) up-regulation of *CDKN1A* and *IGFBP7*, (b) up-regulation of *CDKN2A, IL6* and *IL8* and down-regulation of *CTCF* in pilocytic astrocytomas. Relative expression shown as Log_2_ fold change compared to normal adult cerebellum and frontal lobe (normalized to *TBP*). Data represent two technical replicates ± SD. (ZIP 275 kb)
