# Supplementary material for: Molecular analysis of pediatric brain tumors identifies microRNAs in pilocytic astrocytomas that target the MAPK and NF-κB pathways
Source: Acta Neuropathol Commun. 2015 Dec 18;3:86. doi: 10.1186/s40478-015-0266-3 (PMC4683939; doi:10.1186/s40478-015-0266-3)
Supplement: Additional file 5: Table S5. — (a) List of primer pairs selected for Illumina microRNA expression array (MI-v2) validation. (b) List of primer pairs used to identify KIAA1549-BRAF fusion and the BRAF V600E mutation. (PDF 58 kb) [file 40478_2015_266_MOESM5_ESM.pdf]

a.

Gene	Forward Primer	Reverse Primer	Exons	Size
IGFBP7	GCGAGCAAGGTCCTTCCATA	GGGATTCCGATGACCTCACA	1-2	92bp
CDKN2A (p16)	CCAACGCACCGAATAGTTACG	GCGCTGCCCATCATCATG	1-2	57bp
IL6	AATTCGGTACATCCTCGACGG	TTGGAAGGTTCAGGTTGTTTTCT	2-3	111bp
IL8	AAGGAAAACTGGGTGCAGAG	ATTGCATCTGGCAACCCTAC	3-4	176bp
CDKN1A (p21)	TGTCCGTCAGAACCCATGC	AAAGTCGAAGTTCCATCGCTC	1-2	138bp
CTCF	GAGAAGCCATTCAAGTGTTCCAT	CTCCAGTATGAGAGCGAATGTGA	5-6	85bp
TBP	CACGAACCACGGCACTGATT	TTTTCTTGCTGCCAGTCTGGAC	5-6	88bp

b.

Primer	Primer Sequence
***KIAA1549-BRAF* fusion primers**	
KIAA1549 nested 1 exon 12	GAGGGACGCAGGAGATAAGA
KIAA1549 nested 2 exon 14-15	CCAGGAAGAGCTCACGGATA
BRAF nested 1 exon 13-14	AAGTAATCCATGCCCTGTGC
BRAF nested 2 exon 12	TGCTGAGGTGTAGGTGCTGT
***BRAF* V600E - sequencing**	
BRAFV600E F exon 15	TGCTTGCTCTGATAGGAAAATG
BRAFV600E R exon 15	CCACAAAATGGATCCAGACA
