# Supplementary material for: Molecular analysis of pediatric brain tumors identifies microRNAs in pilocytic astrocytomas that target the MAPK and NF-κB pathways
Source: Acta Neuropathol Commun. 2015 Dec 18;3:86. doi: 10.1186/s40478-015-0266-3 (PMC4683939; doi:10.1186/s40478-015-0266-3)
Supplement: Additional file 6: Table S6. — Ingenuity Pathway Analysis of differentially expressed genes in pilocytic astrocytomas compared to normal adult cerebellum, showing transcription factors with a predicted activation state as (a) ‘activated’ and (b) ‘inhibited’. (PDF 68 kb) [file 40478_2015_266_MOESM6_ESM.pdf]

a.

Upstream Regulator	Activation z-score	p-value overlap
NFkB (complex)	7.008	4.73E-22
TP53	6.166	7.68E-42
IRF7	5.953	6.69E-17
STAT3	5.916	1.71E-34
SP1	5.785	1.11E-16
CTNNB1	5.498	5.10E-26
STAT4	5.333	5.87E-09
HIF1A	5.22	1.65E-12
STAT1	5.157	5.10E-24
SMAD3	5.062	5.21E-16
SIM1	4.99	6.76E-06
ARNT2	4.796	3.75E-05
MTPN	4.42	3.36E-11
GLI1	4.417	1.58E-09
MITF	4.361	9.92E-06
CEBPB	4.308	5.98E-24
IRF1	4.287	1.55E-15
TP73	4.118	1.09E-09
IRF3	4.11	7.27E-13
NFKB1A	4.057	1.63E-29
BRCA1	4.016	4.25E-08
ERG	3.999	1.57E-08
JUN	3.872	7.93E-21
RELA	3.811	7.04E-14
ETS1	3.757	1.80E-08
CREB1	3.754	1.97E-10
PGR	3.737	1.60E-12
CREBBP	3.625	6.29E-09
NFATC2	3.583	5.11E-10
SMAD4	3.565	9.33E-17
Ap1	3.361	8.30E-08
IFI16	3.329	2.71E-06
SMAD1	3.273	4.63E-10
STAT	3.25	2.17E-08
CEBPA	3.191	4.55E-18
EP300	3.133	1.79E-06
SRF	3.127	8.36E-06
EPAS1	3.073	4.09E-07
FOXO3	3.07	7.18E-09
IRF5	3.044	1.16E-06
SMAD2	2.97	2.39E-06
REL	2.874	1.67E-07
NFE2L2	2.854	4.53E-05
KLF4	2.823	4.72E-08

SP1	2.816	8.67E-13
TFEB	2.804	4.18E-05
SMARCB1	2.801	2.58E-05
NFKB1	2.788	4.71E-10
Smad2/3-Smad4	2.779	2.22E-07
SMARCA4	2.761	2.61E-24
EGR1	2.731	7.18E-09
HTT	2.714	8.34E-09
NR3C2	2.678	9.98E-06
T, brachyury homolog	2.646	3.69E-06
SMARCD3	2.646	1.41E-05
ETS2	2.639	1.80E-07
STAT2	2.604	6.00E-08
PML	2.538	2.43E-08
GATA	2.52	2.03E-10
WT1	2.469	9.34E-06
ZBTB17	2.433	1.02E-06
Notch	2.395	1.71E-12
NLRC5	2.364	2.54E-11
NFYA	2.36	9.89E-09
AR	2.34	1.04E-10
Smad	2.271	4.39E-08
MKL1	2.216	6.45E-07
FOXO4	2.204	1.00E-05
TCF3	2.196	1.29E-05
ELK1	2.119	5.53E-09
CIITA	2.075	5.25E-11
STAT5A	2.024	2.27E-06

b.

**Upstream Regulator**	**Activation z-score**	***p*-value overlap**
TRIM24	-4.737	3.00E-12
SMAD7	-3.927	1.49E-15
HOXA10	-3.909	6.66E-11
SPDEF	-3.748	8.50E-13
MYCN	-3.721	1.71E-09
IKZF1	-3.459	2.51E-07
NKX2-3	-3.297	2.95E-23
estrogen receptor	-3.128	1.86E-22
GFI1	-3.052	5.12E-07
ID3	-2.982	5.58E-05
KLF2	-2.913	1.31E-18
ZNF217	-2.891	1.30E-09
MYC	-2.742	9.13E-35
HDAC1	-2.595	1.79E-06

Nr1h	-2.503	5.55E-08
PCGF2	-2.367	1.32E-04
NEUROG1	-2.309	2.16E-05
IRF4	-2.274	1.21E-04
GLIS2	-2.219	6.24E-06
CNOT7	-2.213	1.91E-09
NAB2	-2.213	8.42E-04
RUNX3	-2.213	1.94E-02
ZFP36	-2.202	3.36E-03
ID2	-2.200	2.35E-03
Rb	-2.177	1.03E-02
TSC22D3	-2.157	3.22E-05
HES1	-2.003	3.51E-05
MNT	-2.000	1.10E-03
