# Supplementary figures and images for: Molecular analysis of pediatric brain tumors identifies microRNAs in pilocytic astrocytomas that target the MAPK and NF-κB pathways
Source: Acta Neuropathol Commun. 2015 Dec 18;3:86. doi: 10.1186/s40478-015-0266-3 (PMC4683939; doi:10.1186/s40478-015-0266-3)

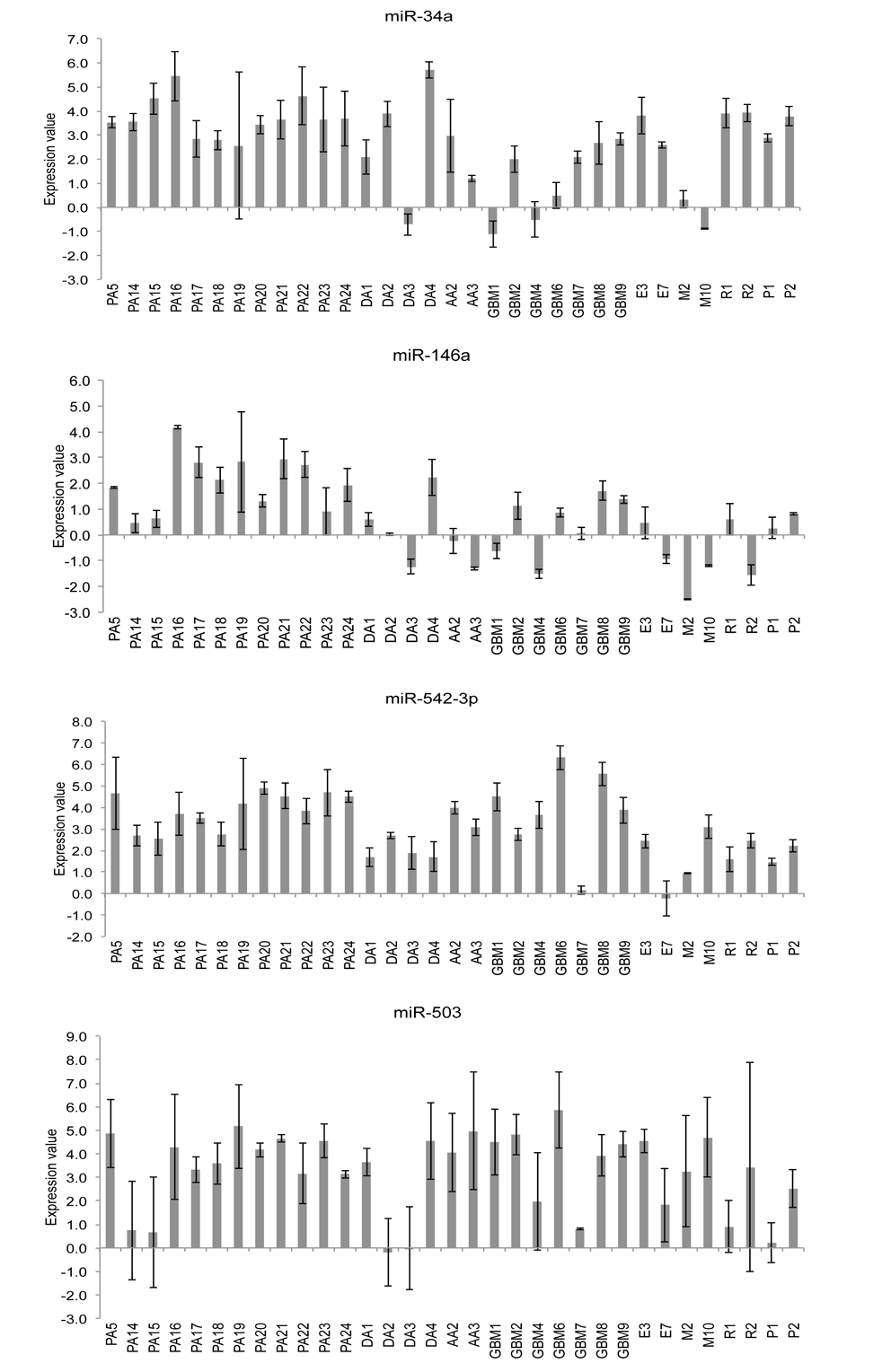

Supplement: Additional file 7: Figure S1. — RT-qPCR confirms (a) up-regulation of miR-34a, miR-146a, miR-542-3p and miR-503 in pilocytic astrocytomas. (b) low expression of miR-124*, miR-129 and miR-129* in pilocytic astrocytomas. Relative expression shown as Log2 fold change compared to normal adult cerebellum and frontal lobe (normalized to miR-423-3p). Data represent two technical replicates ± SD. (ZIP 516 kb) [file 40478_2015_266_MOESM7_ESM.zip › Figure S1a.tif]

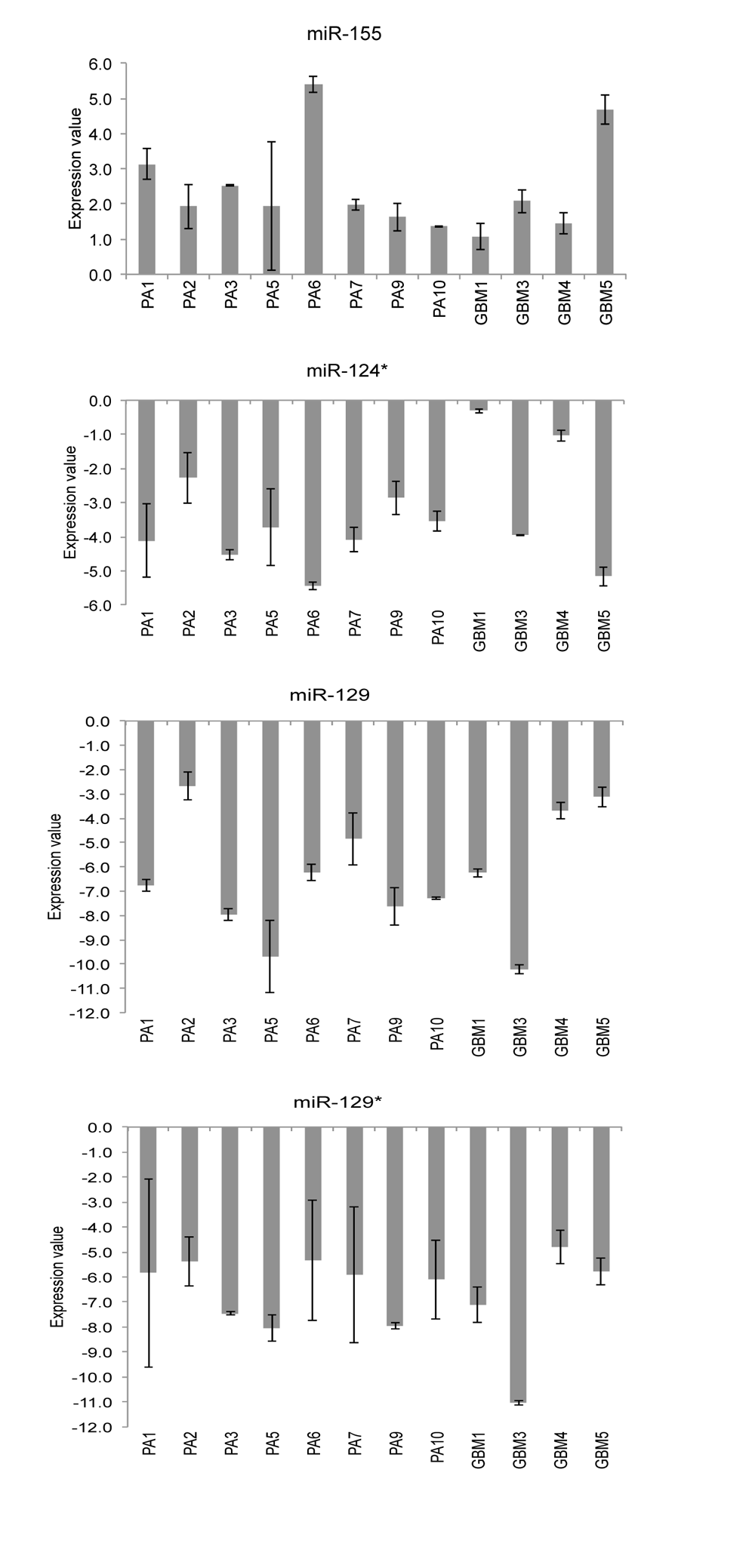

Supplement: Additional file 7: Figure S1. — RT-qPCR confirms (a) up-regulation of miR-34a, miR-146a, miR-542-3p and miR-503 in pilocytic astrocytomas. (b) low expression of miR-124*, miR-129 and miR-129* in pilocytic astrocytomas. Relative expression shown as Log2 fold change compared to normal adult cerebellum and frontal lobe (normalized to miR-423-3p). Data represent two technical replicates ± SD. (ZIP 516 kb) [file 40478_2015_266_MOESM7_ESM.zip › Figure S1b.tif]

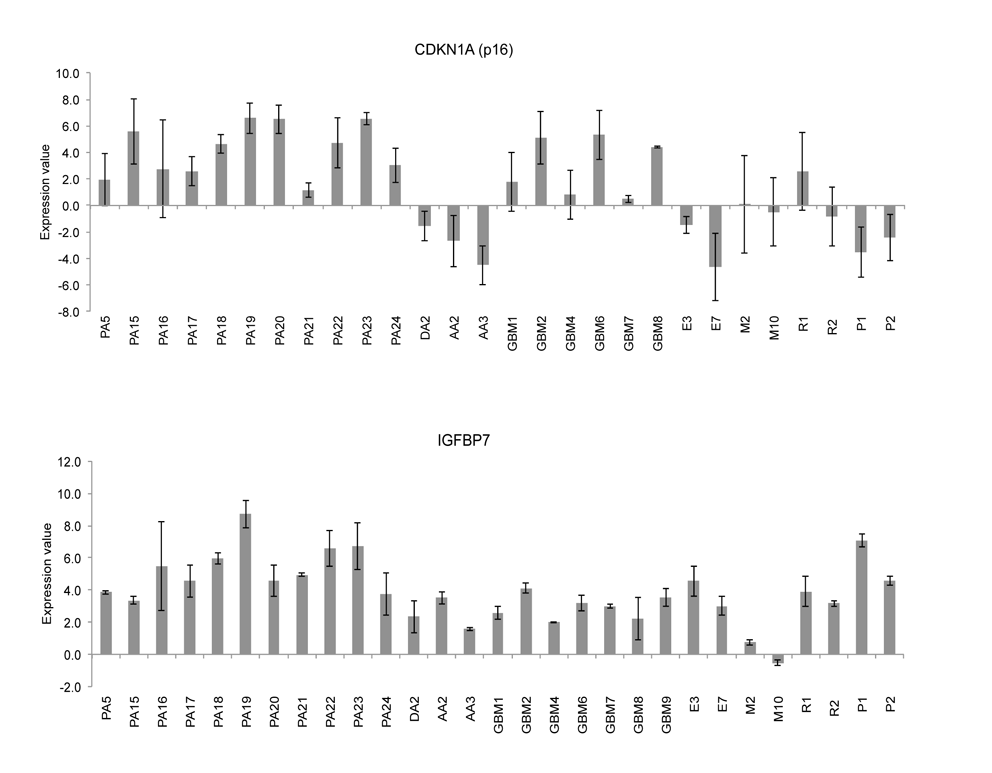

Supplement: Additional file 8: Figure S2. — RT-qPCR confirms (a) up-regulation of CDKN1A and IGFBP7, (b) up-regulation of CDKN2A, IL6 and IL8 and down-regulation of CTCF in pilocytic astrocytomas. Relative expression shown as Log2 fold change compared to normal adult cerebellum and frontal lobe (normalized to TBP). Data represent two technical replicates ± SD. (ZIP 275 kb) [file 40478_2015_266_MOESM8_ESM.zip › Figure S2a.tif]

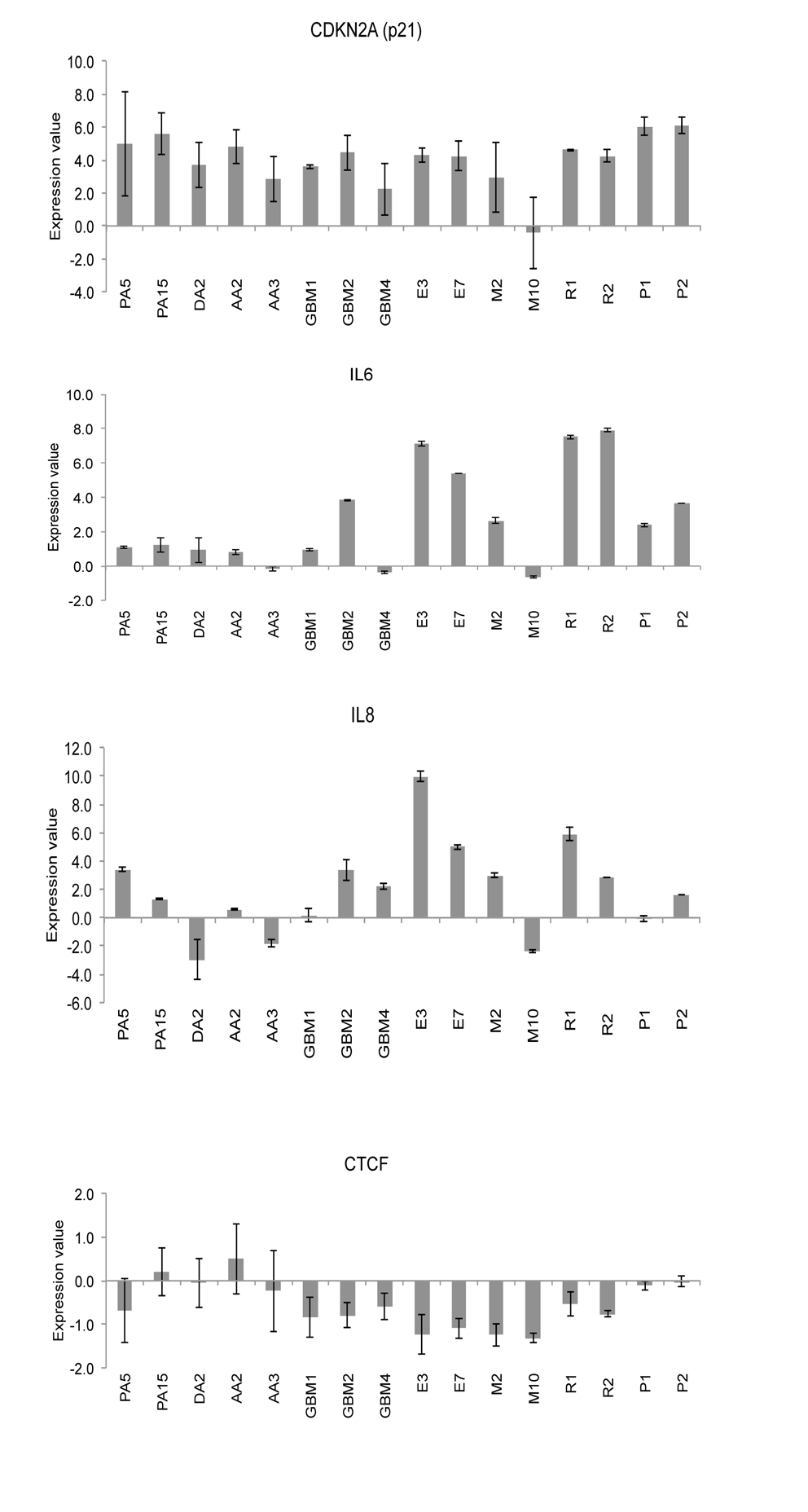

Supplement: Additional file 8: Figure S2. — RT-qPCR confirms (a) up-regulation of CDKN1A and IGFBP7, (b) up-regulation of CDKN2A, IL6 and IL8 and down-regulation of CTCF in pilocytic astrocytomas. Relative expression shown as Log2 fold change compared to normal adult cerebellum and frontal lobe (normalized to TBP). Data represent two technical replicates ± SD. (ZIP 275 kb) [file 40478_2015_266_MOESM8_ESM.zip › Figure S2b.tif]
